# Second primary colorectal cancer among endometrial cancer survivor: shared etiology and treatment sequelae

**DOI:** 10.1007/s00432-018-2599-3

**Published:** 2018-02-14

**Authors:** Myong Cheol Lim, Young-Joo Won, Jiwon Lim, Sang-Soo Seo, Sokbom Kang, Chong Woo Yoo, Joo-Young Kim, Jae Hwan Oh, Robert E. Bristow, Sang-Yoon Park

**Affiliations:** 1 Cancer Healthcare Research Branch, Research Institute, National Cancer Center, Goyang, Republic of Korea; 2 Precision Medicine Branch, Research Institute, National Cancer Center, Goyang, Republic of Korea; 3 Common Cancer Branch, Research Institute, National Cancer Center, Goyang, Republic of Korea; 4 Center for Uterine Cancer, Hospital, National Cancer Center, Goyang, Republic of Korea; 5 Center for Colorectal Cancer, Hospital, National Cancer Center, Goyang, Republic of Korea; 6 Center for Clinical Trials, Hospital, National Cancer Center, Goyang, Republic of Korea; 7 Cancer Registration and Statistics Branch, National Cancer Control Institute, National Cancer Center, Goyang, Republic of Korea; 8 Department of Cancer Control and Population Health, Graduate School of Cancer Science and Policy, National Cancer Center, Goyang, Republic of Korea; 9 Department of Cancer Biomedical Science, Graduate School of Cancer Science and Policy, National Cancer Center, Goyang, Republic of Korea; 10 Division of Gynecologic Oncology, Obstetrics and Gynecology, Irvine Medical Center, University of California, California, USA

**Keywords:** Second primary tumor, Endometrial cancer, Colon cancer, Hereditary, Korea, Lynch syndrome

## Abstract

**Purpose:**

To evaluate the incidence of colon cancer as a second primary cancer (CCSPC) and the survival outcomes of women with and without CCSPC after the diagnosis of endometrial cancer (EC).

**Methods:**

The standardized incidence ratio (SIR) of CCSPC and survival outcomes of EC survivors with and without CCSPC were analyzed using data from January 1 1993 to December 31 2011, obtained from the Korea Central Cancer Registry.

**Results:**

Of 14,797 EC survivors, 147 (0.99%) developed CCSPC after an average interval of 5.5 years. The SIR of CCSPC among EC survivors was 2.56, higher than that of colon cancer in the general population. The SIR of CCSPC was highest for the ascending (3.77), followed by the transverse (3.45), descending colon (2.06), and rectum (1.99). The risk of a proximal site of CCSPC was high, especially within 5 years after the diagnosis of EC in the ascending (SIR, 4.37) and transverse (4.91) colon, and in young survivors (< 60 years) in the ascending (5.19) and transverse (3.82) colon. The 5- and 10-year overall survival rates were 84.8 and 80.4% among survivors with EC only and 89.2 and 76.3% for survivors with CCSPC, respectively.

**Conclusions:**

The risk of CCSPC among EC survivors increases especially in the proximal colon in young survivors. These results could be used for surveillance and counseling of EC survivors.

## Introduction

Endometrial cancer (EC) is an important gynecologic cancer in terms of its incidence and the number of accumulated survivors. In 2016, there were approximately 60,050 new cases of EC in the US, and an estimated 757,190 EC survivors; EC ranks second in terms of the number of women survivors of cancer (Miller et al. 2016). In Korea, the incidence of EC is increasing rapidly, with an annual increase of 6.9% during the period 1999–2010; in 2016 there were 2565 estimated incident cases, and 296 deaths (Lim et al. 2013; Jung et al. 2016).

Second primary cancers have become an issue of concern among survivors of initial primary cancer, especially for whom the initial cancer had a good prognosis, such as EC. Shared etiology such as genetic factors and treatment-related complications should be considered when second primary cancer occurs (Kim et al. 2016). The risk of a second primary cancer after colorectal cancer has been well established according to the anatomic site of the first tumor (Phipps et al. 2013). There is a paucity of research on colon cancer as a second primary cancer (CCSPC) after EC; existing studies have mainly focused on EC as a sentinel cancer of hereditary non-polyposis colorectal cancer syndrome, increased risk of colon cancer after EC and the shared genetic background of mismatch repair gene mutation (Creutzberg et al. 2013; Lu et al. 2005). However, there are no studies regarding the site-specific risk of colon cancer after EC, the risk of CCSPC according to age and the interval after diagnosis of EC, and survival outcomes following EC and CCSPC. Therefore, the objectives of this study were to investigate the incidence of site-specific risk of CCSPC and the survival outcomes of women with and without CCSPC after the diagnosis and treatment of EC.

## Materials and methods

According to data from the Korea Central Cancer Registry (KCCR), from 1993 to 2011, there were 14,797 patients with EC. The KCCR is a nationwide, hospital-based cancer registry that was launched by the Ministry of Health and Welfare in 1980. The KCCR originally collected information on approximately 90% of cancer cases from training hospitals across South Korea. Since 1999, it has been expanded to cover the entire South Korean population under the Population-Based Cancer Registry Program. The incidences of all cancers are recorded by well-trained experts from hospitals annually. The methodology regarding statistical analysis and interpretation of the results of this study is the same as that used in our previous study regarding second primary cancer after cervical cancer (Kim et al. 2016). Standardized incidence ratios (SIRs) and their respective 95% confidence intervals (CIs) were used to quantify the relative risk of second primary cancers among EC survivors compared with women in the general population. These SIRs were calculated by dividing the observed by the expected number of secondary cancers if the patients in the cohort demonstrated cancer rates equivalent to those for individuals in the general population. The number of person-years at risk (PYRs) was defined from 2 months after the date of EC diagnosis to the date of death or the end of this study (December 31, 2011), whichever occurred first. For each initial cancer site grouping, the PYRs and observed cases of cancer were stratified by 5-year age groups and calendar year. Cancer incidence rates were computed for each subsite of cancer according to age and calendar year and multiplied by the accumulated PYRs to estimate the expected number of subsequent cancers for each stratum. Kaplan–Meier survival curves were generated for EC survivors with or without a second cancer. The differences between groups were assessed using the log-rank test. All statistical tests were two-sided, and significance was set at an alpha level of 0.05. To compute the SIRs and their 95% CIs, we used the “MP-SIR” setting of the Surveillance Research Program, National Cancer Institute SEER*Stat software version 8.1.2. (seer.cancer.gov/seerstat). Stata Statistical Software (Release 11; StataCorp LP College Station, TX, USA) was used to generate the survival curves and perform log-rank tests.

Ethical approval for the research protocol was provided by the Institutional Review Board of the National Cancer Center, Goyang, Korea (NCC2014-0068).

## Results

A total of 14,797 survivors of primary EC were evaluated for a mean follow-up period of 5.5 years; their mean age at the initial diagnosis of EC was 52.2 years (Table 1). The incidence of EC peaked at 50–59 years (36.45%). Of the 14,797 EC survivors, 147 (0.99%) developed CCSPC. The mean (± standard deviation) interval from the initial EC diagnosis to the diagnosis of CCSPC was 5.5 ± 4.17 years, and the mean age at diagnosis of the CCSPC was 58.7 ± 9.26 years.

**Table 1:** Characteristics of patients with endometrial cancer, 1993–2011

Variable	*N* (%) or mean ± SD
Women with endometrial cancer	14,797 (100)
Average follow-up, years	5.5 ± 4.56
Average age at diagnosis of endometrial cancer	52.2 ± 11.54
Age at 1st primary cancers diagnosis, years	
< 30	408 (2.76)
30–39	1558 (10.53)
40–49	3879 (2.21)
50–59	5393 (36.45)
60–69	2449 (16.55)
70–79	940 (6.35)
≥ 80	170 (1.15)
Average interval between first endometrial and second colorectal cancers, years	5.5 ± 4.17
Average age at diagnosis of second colorectal cancer, years	58.7 ± 9.26
Number who developed colorectal cancer as a second primary cancer	147 (0.99)
Number of colorectal cancers as second cancers	138 (0.93)
Number of colorectal cancers as third cancers	9 (0.06)

As shown in Table 2, the overall SIR for CCSPC was 2.56 (95% CI 2.16–3.00). The SIRs were highest for CCSPCs located in the ascending colon (3.77), followed by the transverse colon (3.45), descending colon (2.06), and rectum (1.99). The SIR of CCSPC was higher in women aged < 60 years (3.09, 95% CI 2.54–3.72) than in those aged ≥ 60 years (1.69, 95% CI 1.19–2.33). Among young survivors (age < 60 years), the highest risk site of CCSPC was the ascending colon, with an SIR of 5.19 (95% CI 3.50–7.41).

**Table 2:** Risk of colon cancer as second primary cancer after the diagnosis of endometrial cancer, 1999–2011

Cancer site	Age																	
	< 60 years						≥ 60 years						Total					
	O	E	SIR (O/E)	CI lower	CI upper	EAR	O	E	SIR (O/E)	CI lower	CI upper	EAR	O	E	SIR (O/E)	CI lower	CI upper	EAR
Ascending colon	30	5.78	5.19*	3.50	7.41	3.69	8	4.30	1.86	0.80	3.67	2.38	38	10.07	3.77*	2.67	5.18	3.44
Transverse colon	9	2.36	3.82*	1.74	7.24	1.01	5	1.70	2.95	0.96	6.88	2.12	14	4.06	3.45*	1.89	5.79	1.22
Descending colon	24	9.64	2.49*	1.60	3.70	2.19	6	4.96	1.21	0.44	2.64	0.67	30	14.60	2.06*	1.39	2.93	1.90
Rectum	38	15.90	2.39*	1.69	3.28	3.37	12	9.25	1.30	0.67	2.27	1.77	50	25.15	1.99*	1.48	2.62	3.06
Unspecified	9	1.93	4.65*	2.13	8.83	1.08	6	1.71	3.52*	1.29	7.66	2.75	15	3.64	4.12*	2.31	6.80	1.40
Total	110	35.61	3.09*	2.54	3.72	11.33	37	21.9	1.69*	1.19	2.33	9.68	147	57.51	2.56*	2.16	3.00	11.02

	Follow-up duration																	
	2–59 months						60–119 months						120 + months					
	O	E	SIR (O/E)	CI lower	CI upper	EAR	O	E	SIR (O/E)	CI lower	CI upper	EAR	O	E	SIR (O/E)	CI lower	CI upper	EAR
Ascending colon	22	5.04	4.37*	2.74	6.61	3.45	13	3.20	4.06*	2.16	6.94	4.28	3	1.83	1.64	0.34	4.78	1.28
Transverse colon	10	2.03	4.91*	2.36	9.04	1.62	2	1.29	1.55	0.19	5.62	0.31	2	0.73	2.72	0.33	9.84	1.38
Descending colon	13	7.59	1.71	0.91	2.93	1.10	14	4.58	3.05*	1.67	5.12	4.11	3	2.42	1.24	0.26	3.62	0.63
Rectum	26	13.37	1.94*	1.27	2.85	2.57	13	7.80	1.67	0.89	2.85	2.27	11	3.98	2.77*	1.38	4.95	7.68
Unspecified	9	1.91	4.71*	2.15	8.93	1.44	2	1.13	1.77	0.21	6.39	0.38	4	0.60	6.71*	1.83	17.18	3.72
Total	80	29.95	2.67*	2.12	3.32	10.18	44	18	2.44*	1.78	3.28	11.35	23	9.56	2.41*	1.52	3.61	14.69

*SIR* standardized incidence ratio, *O/E* ratio of observed number of second primary cancers to the number of expected cancers, *CI* confidence interval, *EAR* excess absolute risk

*Significantly increased or decreased risk (*P* < 0.05)

Within 5 years after the diagnosis of EC, the overall SIR was 2.67 (95% CI 2.12–3.32). The highest SIR (4.91, 95% CI 2.36–9.04) was found among patients with tumors of the transverse colon, followed by those with tumors of the ascending colon (SIR, 4.37; 95% CI 2.74–6.61) and the rectum (SIR, 1.94; 95% CI 1.27–2.85). Within 6–10 years of follow-up after the diagnosis of EC, the overall SIR was 2.44 (95% CI 1.78–3.28), with the highest SIR among patients with tumors of the ascending colon (4.06, 95% CI 2.16–6.94). More than 10 years after the diagnosis of EC, the overall SIR was 2.41 (95% CI 1.52–3.61), but during this period only that of rectal cancer increased (SIR, 2.77; 95% CI 1.38–4.95), the SIRs of tumors in the ascending, transverse, and descending colon did not. Among EC survivors who received radiation therapy, the risk of rectal second primary cancer increased (SIR, 3.59; 95% CI 1.96–6.03) and was highest > 10 years after the initial diagnosis of EC (SIR, 7.13; 95% CI 1.47–20.83) (Table 3).

**Table 3:** Risk of colon cancer as second primary cancer in radiation treatment group of endometrial cancer, 1999–2011

Cancer site	Follow-up duration																							
	2–59 months						60–119 months						120 + months						Total					
	O	E	SIR (O/E)	CI Lower	CI Upper	EAR	O	E	SIR (O/E)	CI Lower	CI Upper	EAR	O	E	SIR (O/E)	CI Lower	CI Upper	EAR	O	E	SIR (O/E)	CI Lower	CI Upper	EAR
Ascending colon	7	0.93	7.55*	3.04	15.6	8.09	4	0.47	8.43*	2.30	21.60	12.10	1	0.20	5.01	0.13	27.90	9.09	12	1.60	7.50*	3.87	13.10	9.20
Transverse colon	1	0.37	2.69	0.07	15	0.84	0	0.19	0	0	19.40	− 0.65	0	0.08	0	0	46.40	− 0.90	1	0.64	1.56	0.04	8.69	0.32
Descending colon	4	1.36	2.94	0.80	7.52	3.51	6	0.66	9.03*	3.31	19.70	18.30	0	0.26	0	0	14.40	− 2.92	10	2.28	4.38*	2.10	8.06	6.83
Rectum	9	2.37	3.80*	1.74	7.21	8.83	2	1.11	1.80	0.22	6.52	3.06	3	0.42	7.13*	1.47	20.80	29.30	14	3.90	3.59*	1.96	6.03	8.94
Unspecified	1	0.34	2.95	0.07	16.40	0.88	0	0.16	0	0	23.30	− 0.54	1	0.06	15.9	0.4	88.30	10.60	2	0.56	3.57	0.43	12.90	1.27
Total	22	5.37	4.10*	2.57	6.21	22.2	12	2.60	4.62*	2.39	8.07	32.30	5	1.02	4.90*	1.59	11.40	45.20	39	8.98	4.34*	3.09	5.93	26.6

*SIR* standardized incidence ratio, *O/E* ratio of observed number of second primary cancers to the number of expected cancers, *CI* confidence interval, *EAR* excess absolute risk

^*^Significantly increased or decreased risk (*P* < 0.05)

**Fig. 1:**
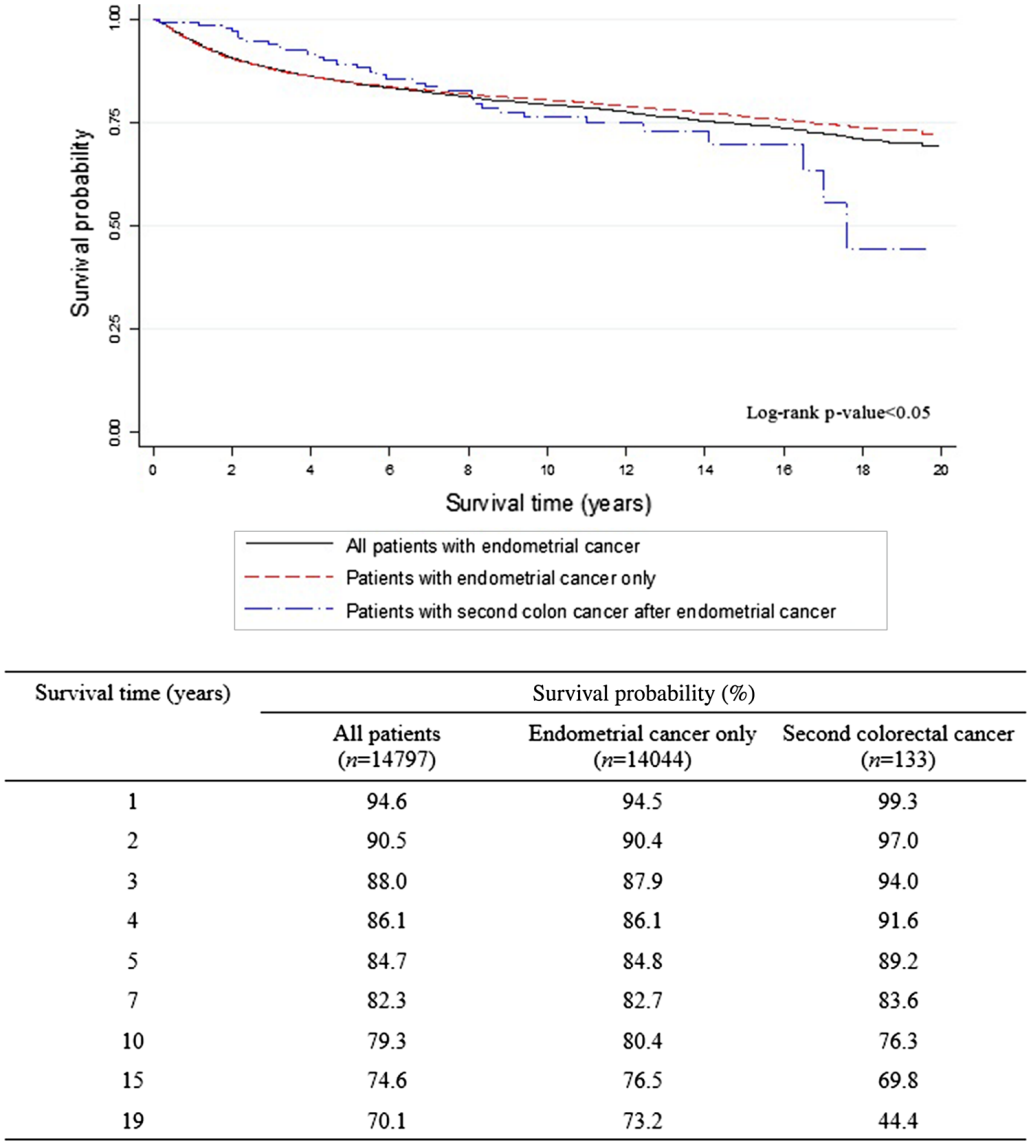
Survival outcomes from the onset of endometrial cancer according to whether CCSPC or not

**Fig. 2:**
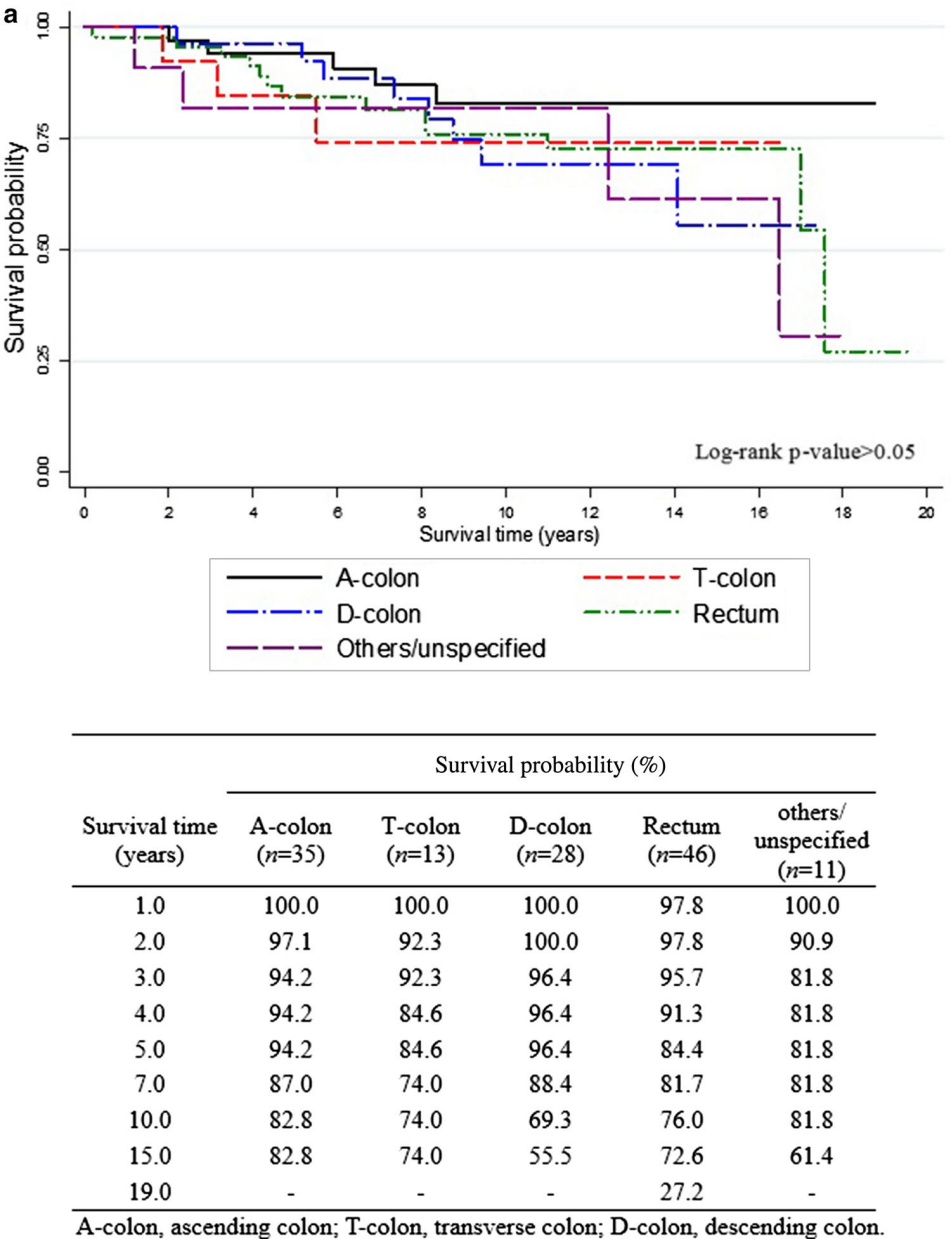

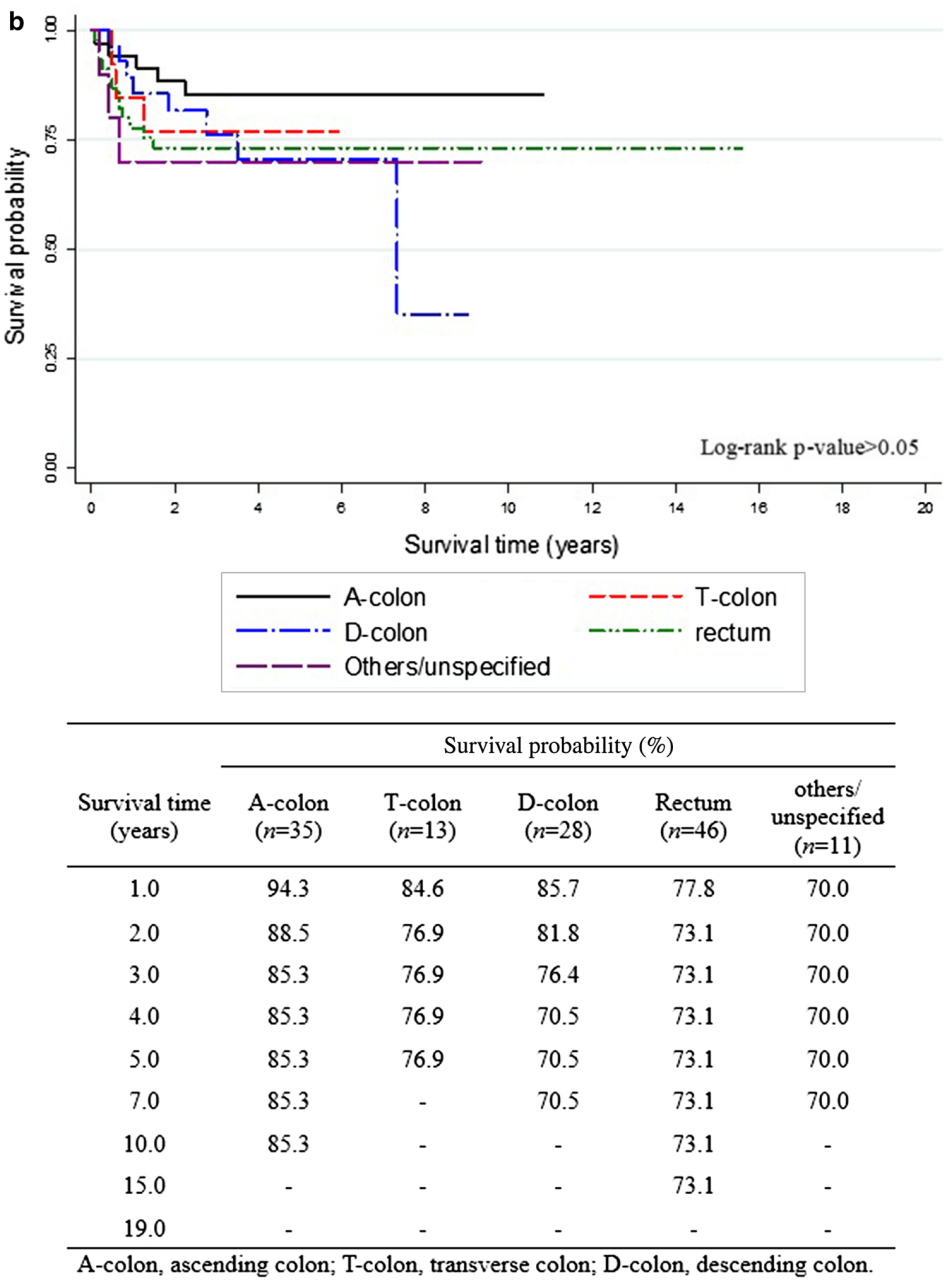
Survival outcomes in endometrial cancer survivors with colon cancer as a second primary cancer. **a** Survival curves from onset of endometrial cancer in endometrial cancer patients with second colon cancer and **b** survival curves from onset of colon cancer as a second primary cancer in survivors of endometrial cancer

The overall 5-year survival rates after the diagnosis of EC (Fig. 1) were 84.7% for all EC survivors, 84.8% for EC survivors without CCSPC, and 89.2% for survivors with CCSPC. The corresponding 10-year overall survival rates were 79.3, 80.4, and 76.3%, respectively. Figure 1 shows that within the first 8 years after the diagnosis of EC, the survival curve for women with CCSPC was more favorable than that of women with EC only; thereafter, the opposite held true. Figure 2 shows survival outcomes according to the site of colon cancer after a diagnosis of EC (Fig. 2a) and CCSPC (Fig. 2b), respectively.

## Discussion

In the current study, the overall SIR of CCSPC in women who survived EC was higher (2.56, 95% CI 2.16–3.00) than the incidence of colon cancer in the general population. Particularly, CCSPC occurred more commonly in the proximal colon than in the distal colon, and in younger (< 60 years) than in older (≥ 60 years) EC survivors. This latter finding suggests the possibility of a shared etiology based on a genetic or environmental background. More than 10 years after the diagnosis of EC, the risk of rectal cancer increased, which might be explained by the sequelae of pelvic radiotherapy for the treatment of primary EC in part. On the other hand, rectal cancer might be suppressed for a period of time with pelvic radiotherapy and then re-emerge. Previously, we reported that most second primary cancers occurring after an initial primary cancer could be explained by shared etiology or a late effect of treatment for the initial primary cancer (Kim et al. 2016). CCSPC in EC survivors may be due to a shared hereditary background for EC. The incidence of EC and colon cancer is increasing at a similar rate in Korea (annual increases of 6.9 and 6.6%, respectively) (Lim et al. 2013; Shin et al. 2012). The more frequent CCSPC in young EC survivors also suggests a hereditary link. A recent publication from a prospective multicenter study revealed that 9% of young patients with EC have germline Lynch syndrome-associated mutations (Lu et al. 2007). The frequency of hereditary non-polyposis colorectal cancer (Lynch syndrome) among Korean EC survivors is significant, as reported in a previous study (Lim et al. 2010). Furthermore, the prevalence of somatic mutations is more frequent than that of germline mutations in patients with endometrial and colon cancers (Haraldsdottir et al. 2014). Further studies are needed to clarify the exact relationship between carcinogenesis due to germline or somatic mutations and environmental factors.

The SIR of CCSPC was highest for tumors situated in the ascending colon. Moreover, the SIR of CCSPC of the ascending colon was 5.19 in young women (aged < 60 years), and 4.37 in EC survivors within 5 years after the diagnosis of EC. The risk of CCSPC was still high (SIR, 4.06) for survivors > 10 years after the diagnosis of EC. In 1995, Lynch et al. reported that young age and proximal colon cancer are characteristic features of Lynch syndrome (Lynch and Lynch 1995). The significant proportion of CCSPC observed among EC survivors in the current study might be explained by a shared etiology of these two cancers, primarily a shared genetic background. An anatomic preference for the lower uterine segment (LUS) as the epicenter of EC has been suggested in cases of hereditary EC (Westin et al. 2008). A greater proportion of women with EC originating in the LUS (29%) have Lynch syndrome than do all EC patients (1.8%) or young EC patients (8–9%) (Westin et al. 2008). Although tumor location in colon cancer has been well investigated and reported, the current cancer registry does not have routine data on the anatomic description of cases of EC. Currently, of the components of the Federation of Gynecology and Obstetrics staging system, only tumor invasion to the cervix has been evaluated and reported. For the evaluation and management of the hereditary component of EC, anatomic evaluation of invasion into the LUS should be evaluated and considered in the cancer registry in the near future. Based on the current results and the interpretation thereof, surveillance for colon cancer should be considered within 10 years after the diagnosis of EC, especially for young women and for those with a tumor epicenter in the LUS. The specific surveillance strategies for the colon cancer in survivors with EC could be specified with the genetic test.

In the current study, while the risk of proximal colon cancer was highest within the first 5 years after the diagnosis of EC, the risk of rectal cancer increased > 10 years after the diagnosis of EC. The increased risk of rectal cancer might be explained by late sequelae of pelvic radiotherapy given to treat primary EC. Rectal bleeding due to rectitis occurs in approximately 18% of women who receive pelvic radiotherapy for EC; it is also a manifestation of the late sequelae of pelvic radiotherapy (Mitra et al. 2015). The risk of rectal cancer was higher (SIR, 1.90, 95% CI 1.74–2.09) among 104,760 cancer survivors (from 13 institutions in 5 countries with more than 40 years of follow-up) who received pelvic radiotherapy, than in the general population. In our previous study of cervical cancer survivors followed-up for a mean period of 7.34 years, the risk of rectal cancer decreased (SIR, 0.74; 95% CI 0.61–0.89) (Kim et al. 2016). However, this early decrease might be explained by a hidden treatment effect of pelvic radiotherapy on microscopic or early rectal cancer. In contrast, the late effect of radiotherapy might increase the risk of secondary cancer, consistent with both the present findings and our previous findings (Mitra et al. 2015). This further supports the need for rectal cancer surveillance among long-term survivors of EC who have undergone pelvic radiotherapy.

In ovarian cancer, the hereditary factor, represented by mutations in *BRCA1* or *BRCA2*, is a strong prognostic factor (Bolton et al. 2012). However, survival outcomes for EC survivors based on hereditary factors—including EC accompanied by colon, ovarian, or breast cancer suggestive of Lynch syndrome; hereditary breast–ovarian cancer syndrome; or Cowden syndrome—are very difficult to analyze because of the high survival rate of EC survivors with these cancers (Yoo et al. 2015). To the best of our knowledge, the previous study performed by our team is the only study published that investigated the impact of the hereditary aspect on survival outcomes in EC survivors (Yoo et al. 2015), although one critical limitation was the small study population that was derived from a single institution. However, in the current study, survival outcomes from a larger study cohort based on data from a national central cancer registry were analyzed. As indicated in Fig. 1, the survival outcome was better for EC survivors without CCSPC than for those with CCSPC based on the overall survival at 8 years after the diagnosis of EC. However, disease-specific survival is not clear; after 8 years, the two survival curves crossed. This may partly be explained by the finding that the frequent somatic mutations found in hereditary EC and colon cancers result in better treatment responses in the early post-diagnosis period (Le et al. 2015). Patients with advanced stage or highly aggressive EC who died relatively quickly would be unable to experience secondary cancer. Nonetheless, the risk of recurrence of both cancers is significant in the late period—after 8 years in this study. On the other hand, long-term sequelae of active anti-cancer treatment, including second primary cancers and morbidities, may also contribute to the observed change in survival curves. These assumptions need to be clarified by further investigations using adequate variables such as genetic test result and disease-specific recurrences. Given the growing push for the mismatch repair screening and/or genetic test in daily clinical practice, genetic information could be abstracted from the medical records of women with EC in future studies.

A strength of the current study is that the results are based on data from the National Central Cancer Registry, which includes all cancers in Korea. Therefore, there is a low possibility of selection bias. Reproducibility is another strength of the current study. The current study cohort has been consistently followed according to the guideline of the Korean Society of Gynecologic Oncology (Lee et al. 2017). This has been feasible and reproducible because of the single-payer insurance system provided by the government in Korea. However, there are also some limitations, including limited clinical information such as weight, radiation, genetic test for Lynch syndrome, disease-specific recurrences which might result in potential flaw in the interpretation of the survival outcome of CCSPC, a relatively short follow-up time, and a relatively infrequent incidence of CCSPC, which limits clearer identification of subgroups at high risk of CCSPC.

In conclusion, the risk of CCSPC is higher among EC survivors than the risk of primary colon cancer in the general female population, especially within the first 5 years after the diagnosis of EC and in young survivors of EC. The preference of CCSPC for the proximal colon and young EC survivors is suggestive of a shared genetic and environmental etiology of endometrial and colon cancers. The increased risk of rectal cancer in long-term EC survivors > 10 years after the diagnosis of EC indicates the possible involvement of sequelae of radiotherapy used to treat EC. Survival outcomes might be quite different according to the development of CCSPC in EC survivors before and after 8 years of the diagnosis of EC. There is a need to study the survival curves across all groups in the near future. These results could be used for surveillance and counseling of EC survivors.

## References

[CR1] Bolton KL, Chenevix-Trench G, Goh C (2012). Association between BRCA1 and BRCA2 mutations and survival in women with invasive epithelial ovarian cancer. CA Cancer J Clin.

[CR2] Creutzberg CL, Kitchener HC, Birrer MJ (2013). Gynecologic cancer intergroup (GCIG) endometrial cancer clinical trials planning meeting: taking endometrial cancer trials into the translational era. Int J Gynecol Cancer.

[CR3] Haraldsdottir S, Hampel H, Tomsic J (2014). Colon and endometrial cancers with mismatch repair deficiency can arise from somatic, rather than germline, mutations. Gastroenterology.

[CR4] Jung KW, Won YJ, Oh CM (2016). Prediction of cancer incidence and mortality in Korea, 2016. Cancer Res Treat.

[CR5] Kim SI, Lim MC, Lee JS (2016). Comparison of lower extremity edema in locally advanced cervical cancer: pretreatment laparoscopic surgical staging with tailored radiotherapy versus primary radiotherapy. Ann Surg Oncol.

[CR6] Le DT, Uram JN, Wang H (2015). PD-1 blockade in tumors with mismatch-repair deficiency. N Engl J Med.

[CR7] Lee SW, Lee TS, Hong DG (2017). Practice guidelines for management of uterine corpus cancer in Korea: a Korean Society of Gynecologic Oncology Consensus Statement. J Gynecol Oncol.

[CR8] Lim MC, Seo SS, Kang S, Seong MW, Lee BY, Park SY (2010). Hereditary non-polyposis colorectal cancer/Lynch syndrome in Korean patients with endometrial cancer. Jpn J Clin Oncol.

[CR9] Lim MC, Moon EK, Shin A (2013). Incidence of cervical, endometrial, and ovarian cancer in Korea, 1999–2010. J Gynecol Oncol.

[CR10] Lu KH, Dinh M, Kohlmann W (2005). Gynecologic cancer as a “sentinel cancer” for women with hereditary nonpolyposis colorectal cancer syndrome. Obstet Gynecol.

[CR11] Lu KH, Schorge JO, Rodabaugh KJ (2007). Prospective determination of prevalence of lynch syndrome in young women with endometrial cancer. J Clin Oncol.

[CR12] Lynch HT, Lynch J (1995). Genetics, natural history, surveillance, management, and gene mapping in the Lynch syndrome. Pathol Biol (Paris).

[CR13] Miller KD, Siegel RL, Lin CC (2016). Cancer treatment and survivorship statistics, 2016. CA Cancer J Clin.

[CR14] Mitra D, Nout R, Catalano PJ (2015). Rectal bleeding after radiation therapy for endometrial cancer. Radiother Oncol.

[CR15] Phipps AI, Chan AT, Ogino S (2013). Anatomic subsite of primary colorectal cancer and subsequent risk and distribution of second cancers. Cancer.

[CR16] Shin A, Kim KZ, Jung KW (2012). Increasing trend of colorectal cancer incidence in Korea, 1999–2009. Cancer Res Treat.

[CR17] Westin SN, Lacour RA, Urbauer DL (2008). Carcinoma of the lower uterine segment: a newly described association with Lynch syndrome. J Clin Oncol.

[CR18] Yoo HJ, Lim MC, Son Y (2015). Survival outcome in endometrial cancer patients according to hereditary predisposition. Taiwan J Obstet Gynecol.

